# Supplementary material for: Targeting vasoactive intestinal peptide-mediated signaling enhances response to immune checkpoint therapy in pancreatic ductal adenocarcinoma
Source: Nat Commun. 2022 Oct 27;13:6418. doi: 10.1038/s41467-022-34242-4 (PMC9613684; doi:10.1038/s41467-022-34242-4)
Supplement: Supplementary file 1 — Supplementary Information [file 41467_2022_34242_MOESM1_ESM.pdf]

Supplementary Information

Targeting vasoactive intestinal peptide-mediated signaling enhances response to immune checkpoint therapy in pancreatic ductal adenocarcinoma

Author list

Sruthi Ravindranathan[1,2*], Tenzin Passang[1,2], Jian-Ming Li[1,2], Shuhua Wang[1,2], Rohan Dhamsania[1,2], Michael Brandon Ware[1,2], Mohammad Y. Zaidi[1,2], Jingru Zhu[1,2], Maria Cardenas[3], Yuan Liu[2,4], Sanjeev Gumber[5,6], Brian Robinson[5], Anish Sen-Majumdar[7], Hanwen Zhang[1], Shanmuganathan Chandrakasan[8], Haydn Kissick[2,3,9], Alan B. Frey[7], Susan N. Thomas[3,10,11], Bassel F. El-Rayes[1,2], Gregory B. Lesinski[1,2], Edmund K. Waller[1,2*].

Affiliations

[1]Department of Hematology and Medical Oncology, Emory University School of Medicine, Atlanta, GA, USA.

[2]Winship Cancer Institute, Emory University, Atlanta, GA, USA.

[3]Department of Urology, Emory University School of Medicine, Atlanta, GA, USA.

[4]Rollins School of Public Health, Emory University, Atlanta, GA, USA.

[5]Department of Pathology and Laboratory Medicine, Emory University School of Medicine, Atlanta, GA, USA.

[6]Yerkes National Primate Research Center, Emory University, Atlanta, GA, USA.

[7]Cambium Oncology LLC, Atlanta, GA, USA.

[8]Aflac Cancer and Blood Disorders Center, Emory University, Atlanta, GA, USA.

[9]Emory Vaccine Centre, Emory University, Atlanta, GA, USA.

[10]Woodruff School of Mechanical Engineering, Georgia Institute of Technology, Atlanta, GA, USA.

[11]Parker H. Petit Institute of Bioengineering and Bioscience, Georgia Institute of Technology, Atlanta, GA, USA.

Corresponding authors

*To whom correspondence should be addressed:

srra@outlook.com and ewaller@emory.edu

Supplementary Figures

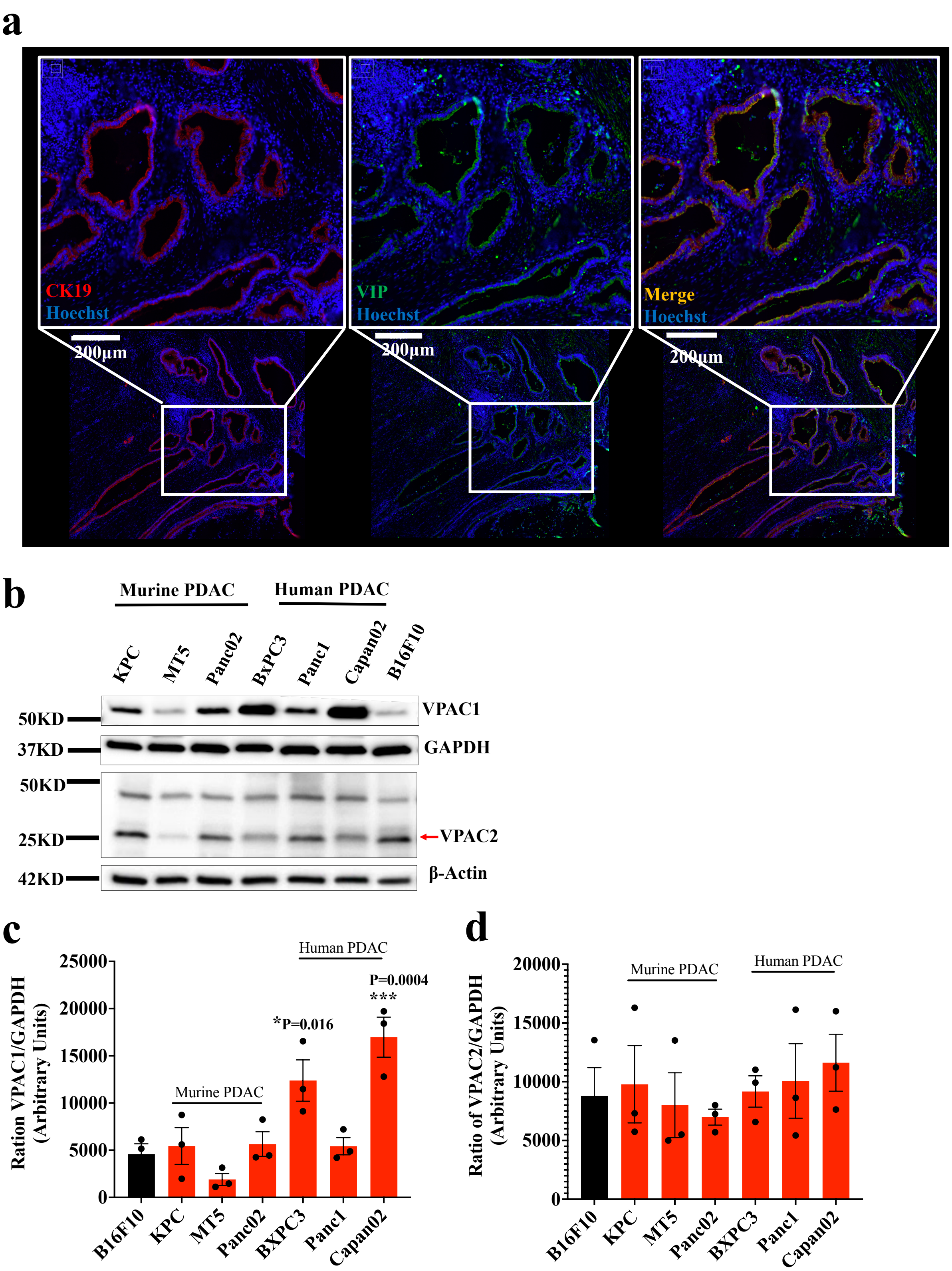

Supplementary Fig. 1. PDAC cell lines and human PDAC tissues express VIP and receptors for VIP. (a) Representative images of one human PDAC tumor stained with antibodies to VIP (green), CK19 (red), and merged (yellow) showing VIP expression in cancer epithelial cells. Scale bars represent 200μm. The experiment was performed once. (b) Representative western blot of lysates from murine melanoma; and murine and human PDAC cell lines probed for VPAC1, VPAC2, and GAPDH as control. The experiment was performed three independent times with similar results. (c) VPAC1, (d) VPAC2 protein bands from western blot were analyzed by densitometric analysis and normalized against the intensity of GAPDH. Results are the mean ± SEM of three independent experiments. P values in c were determined by ANOVA followed by two-tailed Dunnett's post-test. *$p<0.05$, ***$p<0.001$.

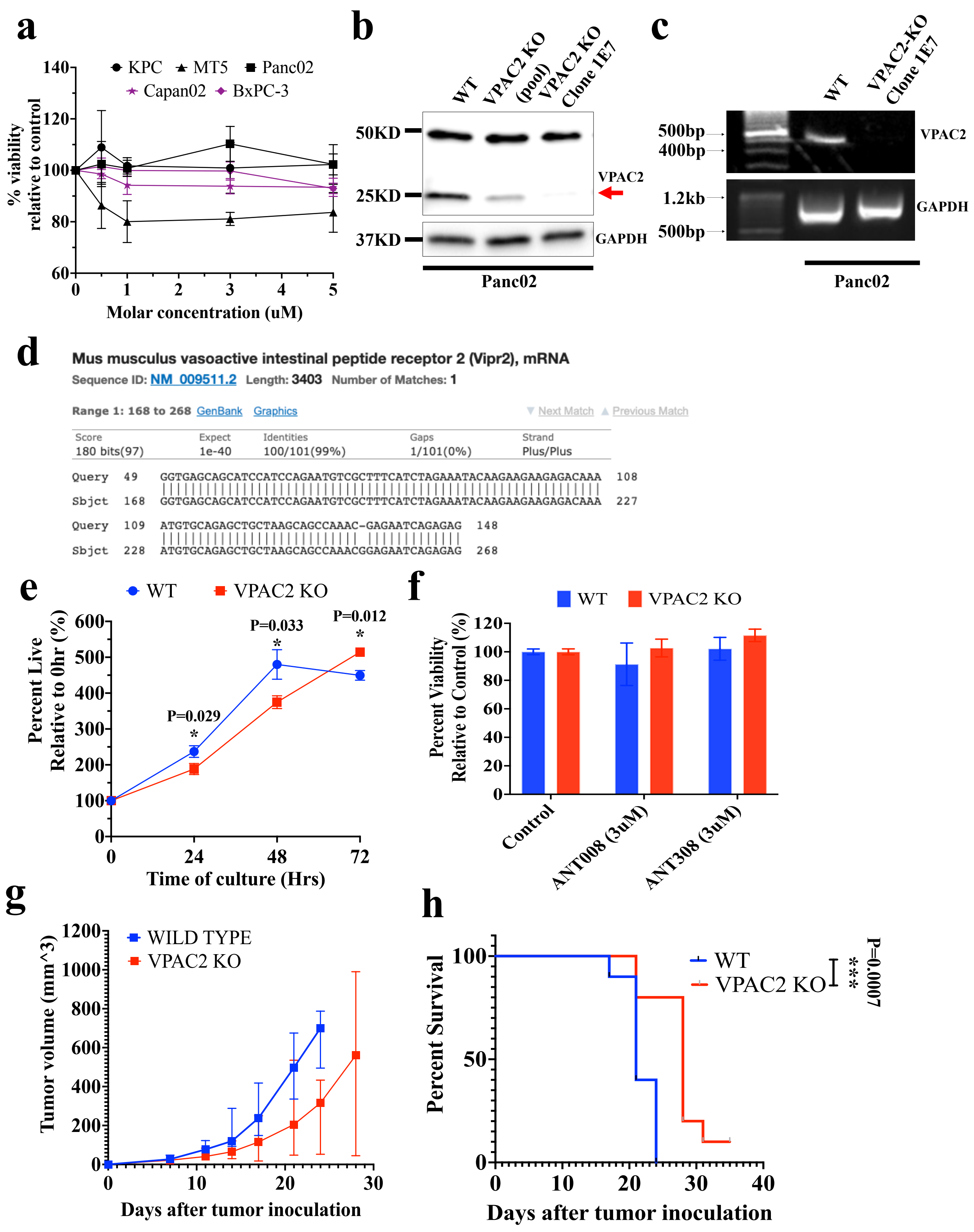

Supplementary Fig. 2. Absence of VPAC2 receptor on PDAC cells confer limited autocrine effect on the growth of cancer cells *in vitro* and *in vivo*. (a) Percentage viability of murine (MT5, KPC.Luc, Panc02) and human (Capan02, BxPC3) PDAC cell

lines cultured in the presence of different concentrations ranging from 0-5μM of ANT008 for 72 hours is plotted. Confirmation of CRISPR-Cas9 KO of *VIPR2* encoding VPAC2 receptor via (b) western blot; Experiment was performed before inoculating cells to mice (c) RT-PCR using primers targeting exon 9-12 downstream of the targeted site. The experiment was done three times with reproducible results; (d) Sanger Sequencing showing the validation of in-del mutation in exon 2. *In vitro* MTT assay showing (e) Proliferation of WT and KO cells over 72 hours; Statistical differences in e were calculated via two-tailed multiple unpaired t-tests with Welch correction. (f) Percent viability of wild type (WT) and VPAC2 KO (KO) Panc02 cells treated with ANT008 and ANT308 at 3μM for 72 hours. (g) Tumor growth curve of WT versus KO Panc02 cells in C57BL/6 mice following subcutaneous tumor implantation (n=10 per group). Values represent median tumor volume ± 95% confidence interval. (h) Kaplan-Meier survival plots corresponding to results in g. The median survival time for WT is 21 days and 28 days for VPAC2 KO. Error bars represent mean and standard deviation. *p<0.05, **p<0.01.

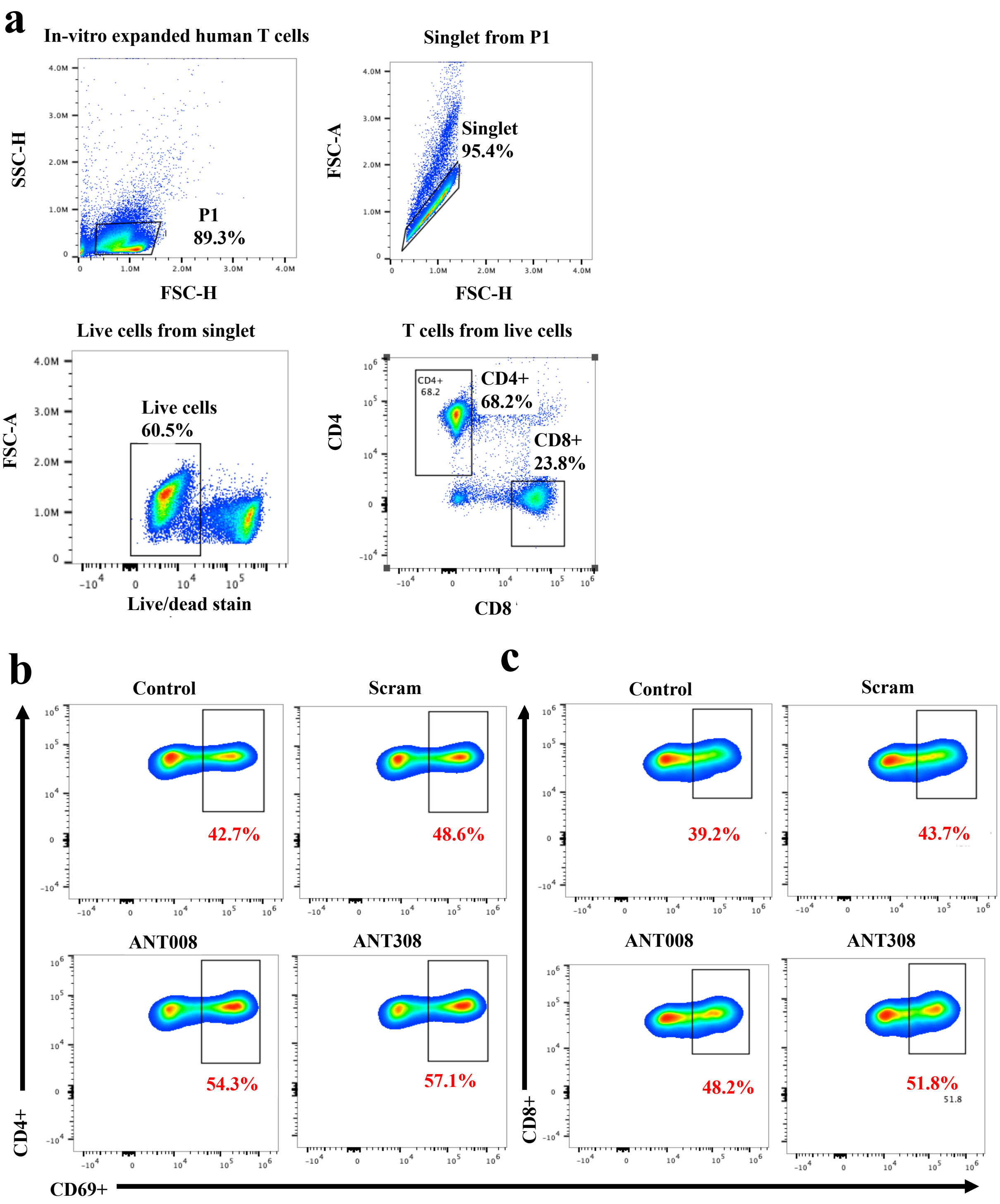

Supplementary Fig. 3. Gating strategy for flow cytometric analysis of healthy human T cells. (a) Cells were gated as ‘P1’ by plotting forward scatter height (FSC-H) and side scatter height (SSC-H). Singlet from P1 was selected by gating along the diagonal on the forward scatter height (FSC-H) versus forward scatter area (FSC-A)

plot. Live cells from singlets were selected by plotting live/dead versus FSC-A. The live cells were then plotted on CD4 versus CD8 plot, to identify CD4+ and CD8+ T cells. CD69 expressing T cells in (b) CD4+ and (c) CD8+ subsets were then identified by plotting each subset on CD4/CD8 versus CD69 flow plots. The percentage of CD69+ T cells within each subset is shown in red.

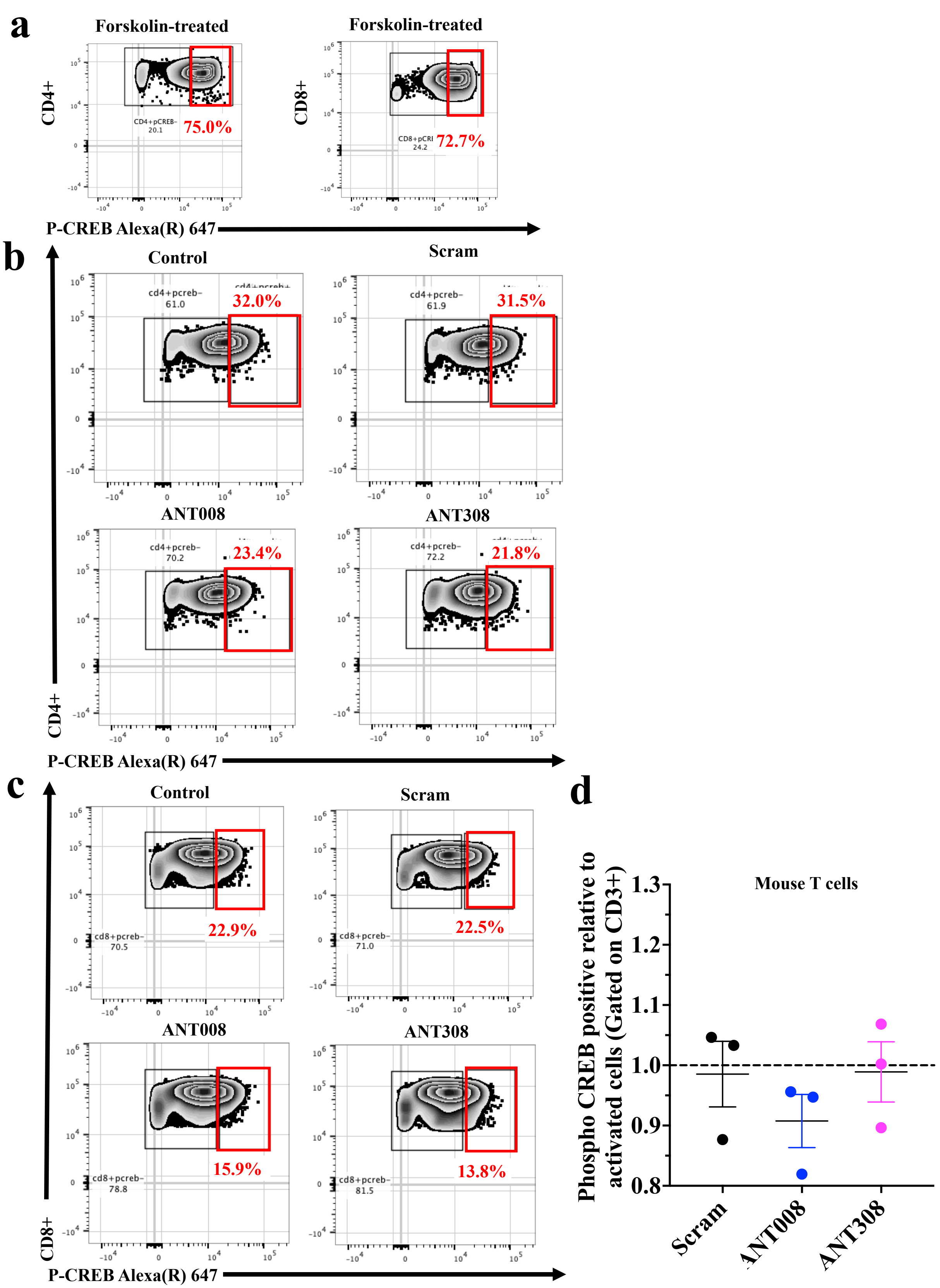

Supplementary Fig. 4. Gating strategy for flow cytometric analysis of CREB phosphorylation in T cells. (a) Plots for Forskolin-treated human T cells used as positive control for gating phospho-CREB positive cells. T cells were treated with

forskolin at 30μM on ice for 30 mins and stained for the surface expression of CD4 and CD8, followed by intracellular staining with anti-phospho-CREB (S133) antibody. Representative plots for phospho-CREB expression in (b) CD4+ and (c) CD8+ human T cells when treated with scrambled peptide (Scram), ANT008, and ANT308 at 3μM for 6h (d) Percentage of CD3+phospho-CREB+ in murine T cells under similar conditions as in c. Error bars show mean ± SEM (n= 3 technical replicates).

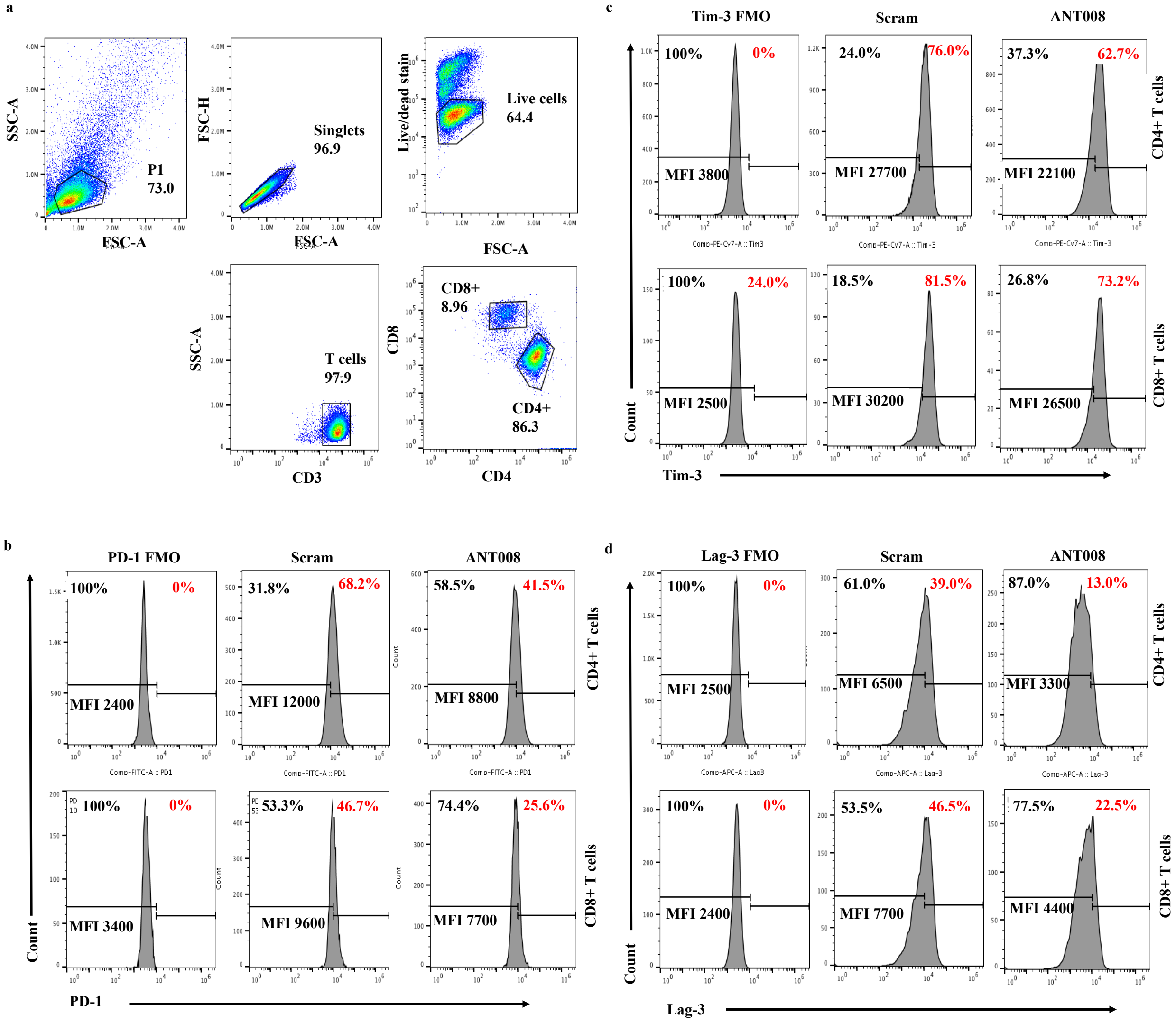

Supplementary Fig. 5. Gating strategy for PD-1, Tim-3 or Lag-3 expression on PDAC patient CD4+ or CD8+ T cells expanded ex-vivo over 9 days. (a) Cells were gated as 'P1' by plotting the forward scatter area (FSC-A) and side scatter area (SSC-A). Singlet from P1 was selected by gating along the diagonal on the forward scatter height (FSC-H) versus forward scatter area (FSC-A) plot. Live cells from singlets were selected by plotting live/dead versus FSC-A. The live cells were then plotted on CD3 versus FSC-A plot, and the cells that are positive for CD3 were gated as T cells. CD4+ and CD8+ T cells were then discriminated by plotting T cells on CD4 versus CD8 plots. (b) PD-1+, (c) Tim-3+ and (d) Lag-3+ cells were gated on CD4+ (top) or CD8+ (bottom) T cells based on FMO controls.

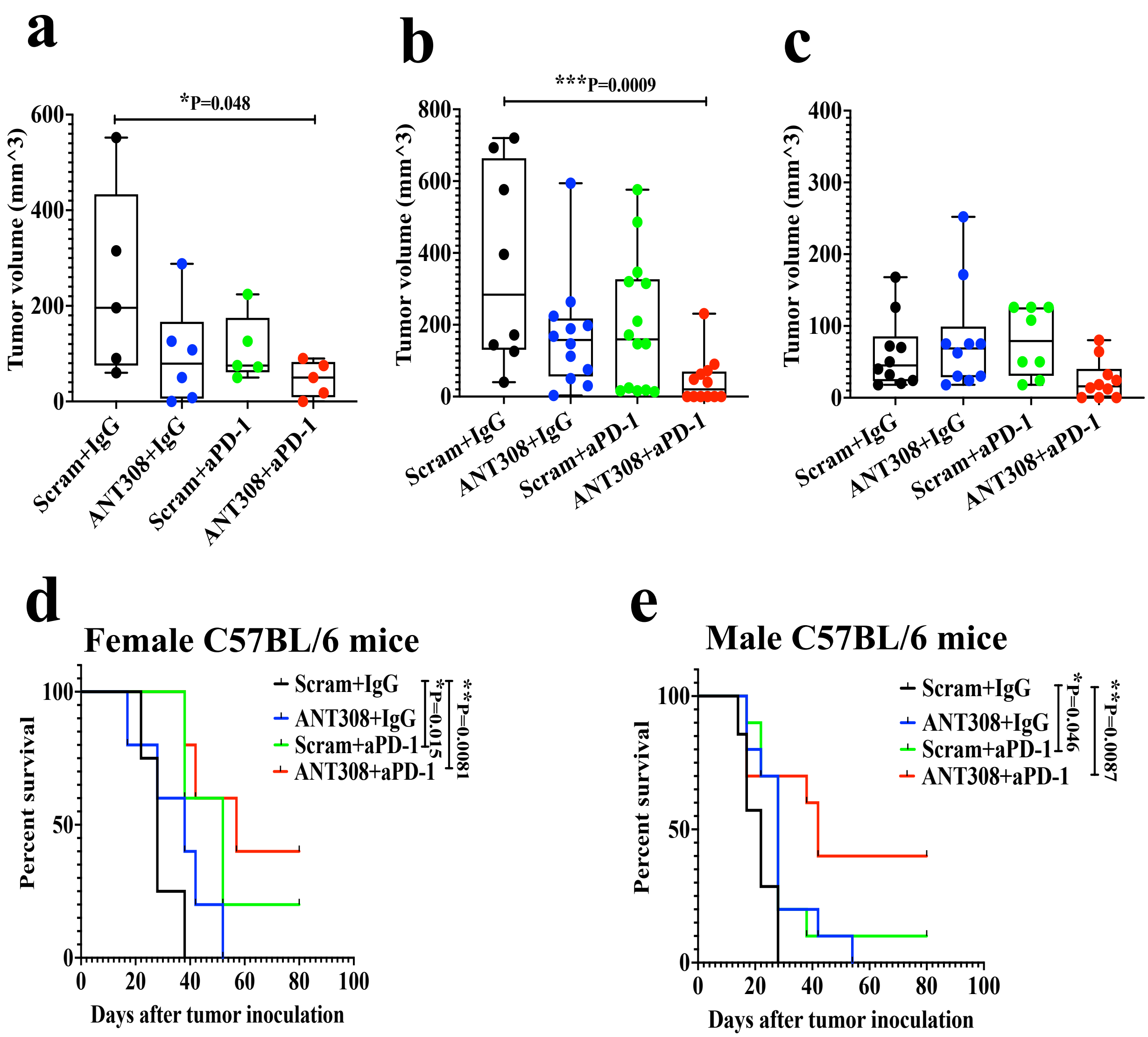

Supplementary Fig. 6. Combination therapy with VIP-R antagonist and anti-PD-1 reduces tumor burden and improves survival in male and female C57BL/6 mice with KPC tumors. Boxplot showing tumor volumes of MT5 (a); KPC-Luc (b) and Panc02 (c) tumor volumes as measured by Vernier calipers on day 22 for MT5 and day 22 for KPC and Panc02 after subcutaneous tumor implantation. Kaplan-Meir survival curve of (d) female (n=5 per group) or (e) male (n=10 per group) C57BL/6 mice subcutaneously implanted with KPC.Luc tumors and treated with ANT308 (female: 10μg, male: 20μg) and/or anti-PD-1. Statistical differences in a-c were calculated by ANOVA followed by two-tailed Dunnett's post-test. The solid line shows the median within each treatment group. Statistical differences in d and e are calculated via the Log-rank test. *p<0.05, **p<0.01 and ***p<0.001.

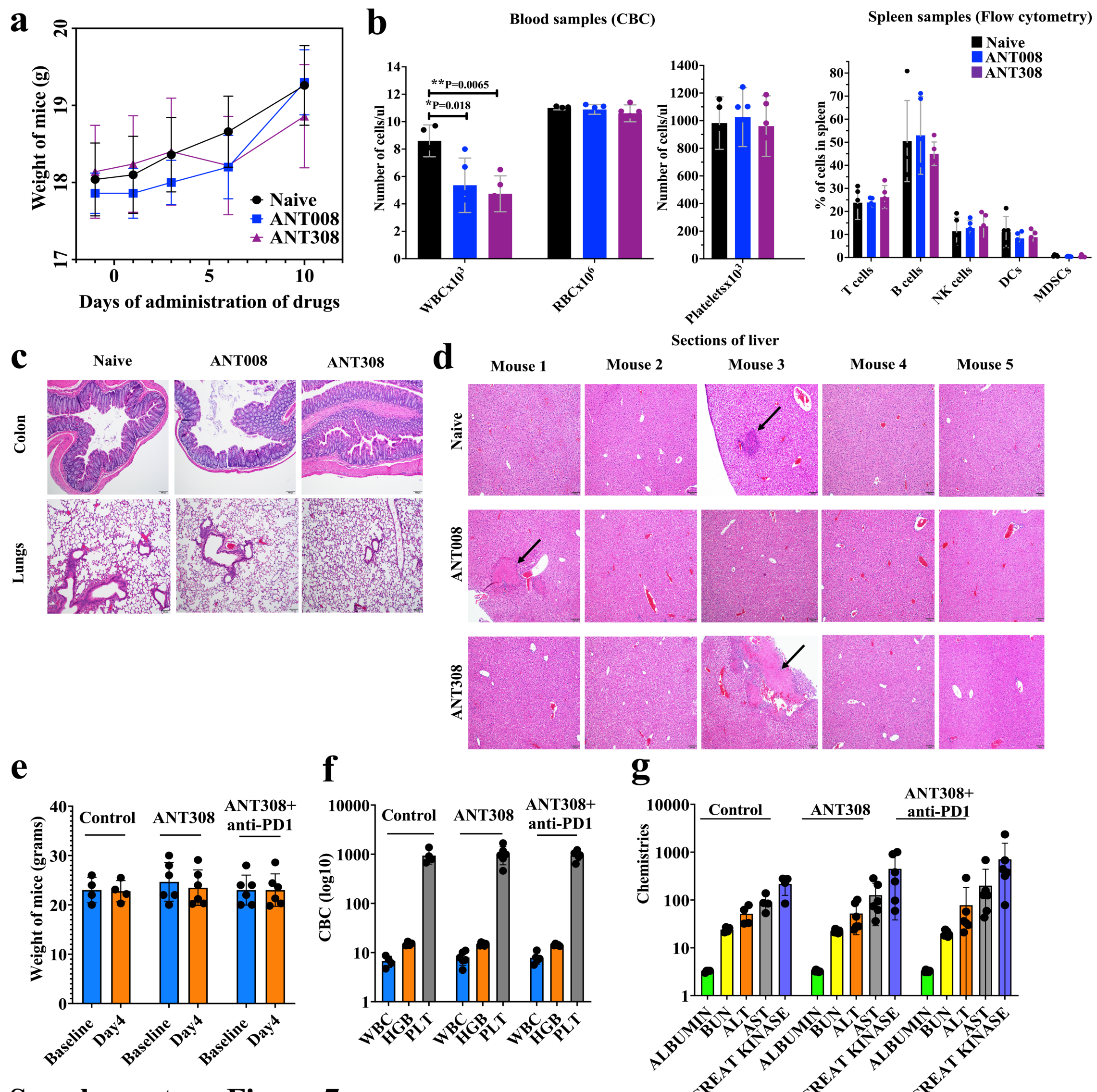

Supplementary Figure 7

Supplementary Fig. 7. Administration of ANT008 or ANT308 showed no adverse toxicity in C57BL/6 mice. C57BL/6 mice received a daily subcutaneous injection of ANT008 or ANT308 for 10 days (n=5 per group) and were analyzed for evidence of toxicity on day 11. (a) Body weight in grams during the duration of drug administration; (b) Number of WBCs, RBCs, and platelets in blood as per complete blood count (left) proportions of T cells, B cells, NK cells, DCs and MDSCs in the spleen as identified by flow cytometry (right) are plotted. Representative H&E-stained sections of (c) colon (top), lungs (bottom), and (d) liver are shown. Arrows in D show focal hepatic lesions in the liver. The focal hepatic necrosis that was observed in one of five mice in each group is not considered drug-related toxicity, as these lesions are commonly observed in several in-bred mice strains at the Jackson Laboratory [1, 2]. C57BL/6 mice received daily subcutaneous injections of 30μg of ANT308 (n=6) or a combination of 30μg of ANT308 daily along with 200μg of anti-PD1 every 3 days (n=6), for a duration of 4 days. Mice receiving scrambled peptide and isotype IgG served as control (n=4). (e) Weight of the mice, (f) complete blood count (CBC), and (g) serum chemistries after 4 days of treatment are plotted. P values in b, e, f, and g were calculated by ANOVA followed by

Dunnett's post-test. Experiments on c and d were performed once with samples from multiple mice. *p*-values in **b** were calculated by ANOVA followed by two-tailed Dunnett's post-test. Error bars represent mean and standard deviation. *p<0.05, **p<0.01.

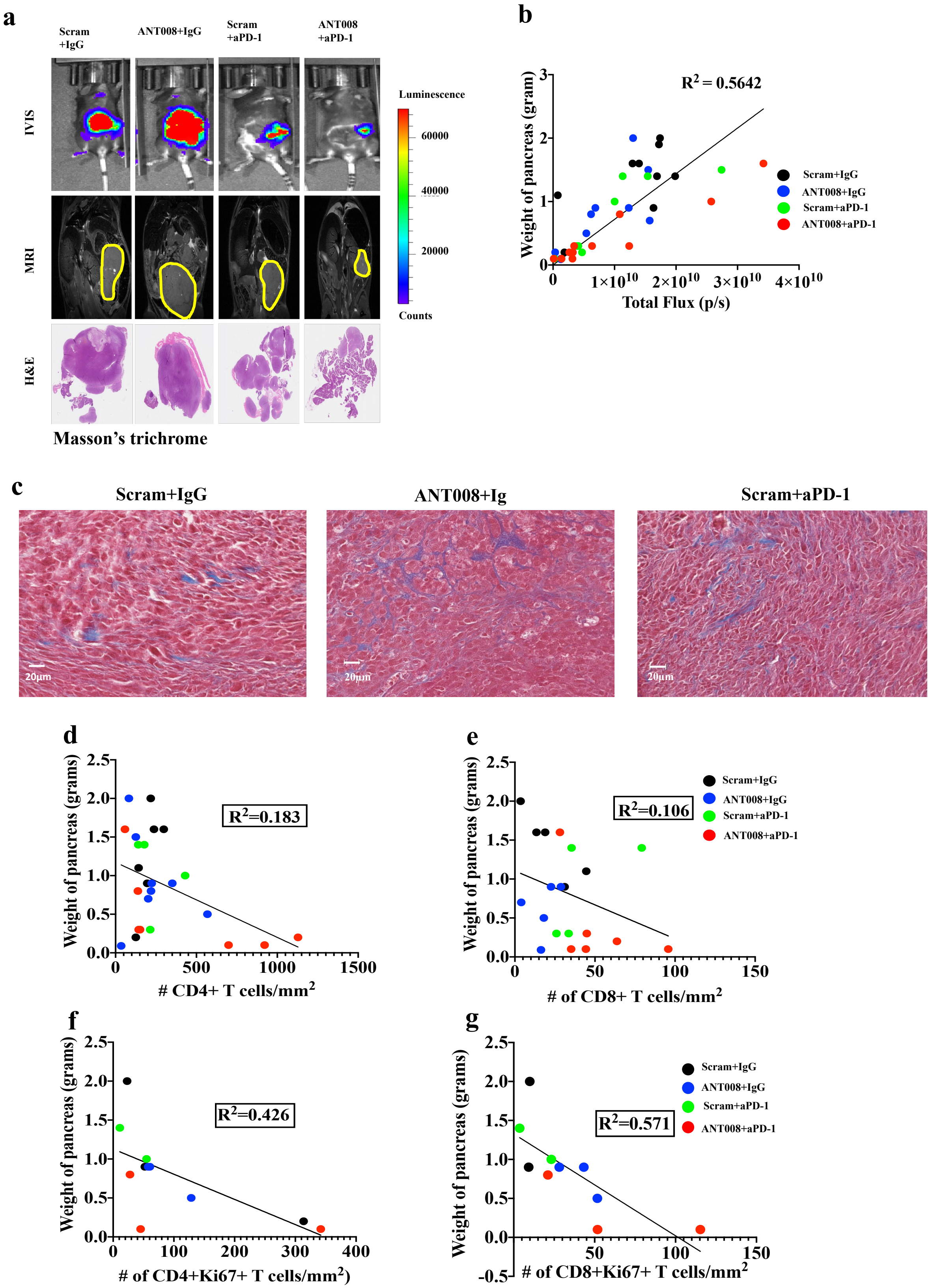
a
Scram +IgG
ANT008+IgG
Scram +aPD-1
ANT008 +aPD-1
IVIS
MRI
H&E
Luminescence
60000
40000
20000
Counts
Masson's trichrome
b
R^2 = 0.5642
Weight of pancreas (gram)
Total Flux (p/s)
0
1×10^10
2×10^10
3×10^10
4×10^10
Scram+IgG
ANT008+IgG
Scram+aPD-1
ANT008+aPD-1
c
Scram+IgG
ANT008+Ig
Scram+aPD-1
20µm
d
R^2=0.183
Weight of pancreas (grams)
CD4+ T cells/mm²
e
R^2=0.106
Weight of pancreas (grams)
# of CD8+ T cells/mm²
Scram+IgG
ANT008+IgG
Scram+aPD-1
ANT008+aPD-1
f
R^2=0.426
Weight of pancreas (grams)
# of CD4+Ki67+ T cells/mm²)
g
R^2=0.571
Weight of pancreas (grams)
# of CD8+Ki67+ T cells/mm²
Scram+IgG
ANT008+IgG
Scram+aPD-1
ANT008+aPD-1

Supplementary Fig. 8. Bioluminescent signal from orthotopically implanted KPC-Luc tumors positively correlated with tumor burden and demonstrates histologic desmoplasia. (a) On day 26 after orthotopic KPC-Luc tumor implantation in C57BL/6 mice, tumor burden in representative mice indicated by 'circle' symbol in Fig. 6c, were compared via bioluminescent imaging, IVIS imaging, and H&E staining of formalin-fixed pancreas isolated after euthanasia. For bioluminescent imaging, isoflurane was used for anesthesia. (b) Total flux (p/s) as measured by bioluminescent imaging on day 26 after tumor implantation was plotted with respect to the weight of the isolated pancreas after euthanasia. Data points are color-coded to represent mice in different treatment groups with n=9, 10, 8, and 11 in scrambled+IgG, ANT008+IgG, scrambled+anti-PD-1, and ANT008+anti-PD-1, respectively. (c) Trichrome staining showing blue collagen stains in the tissue for orthotopically implanted KPC-Luc tumors in all treatment groups. Representative images for scrambled+IgG, ANT008+IgG, scrambled+anti-PD-1 shown; ANT008+anti-PD-1 shown in Figure 6e. Experiments on c were performed once with samples from multiple mice. XY plot showing the correlation, summarized as R-squared, between number of (d) CD4+ or (e) CD8+ T cells/mm^2; and (f) Ki67+ CD4+ or (g) Ki67+ CD8+ T cells/mm^2 with weight of the pancreas with n=4 to 6 mice per group.

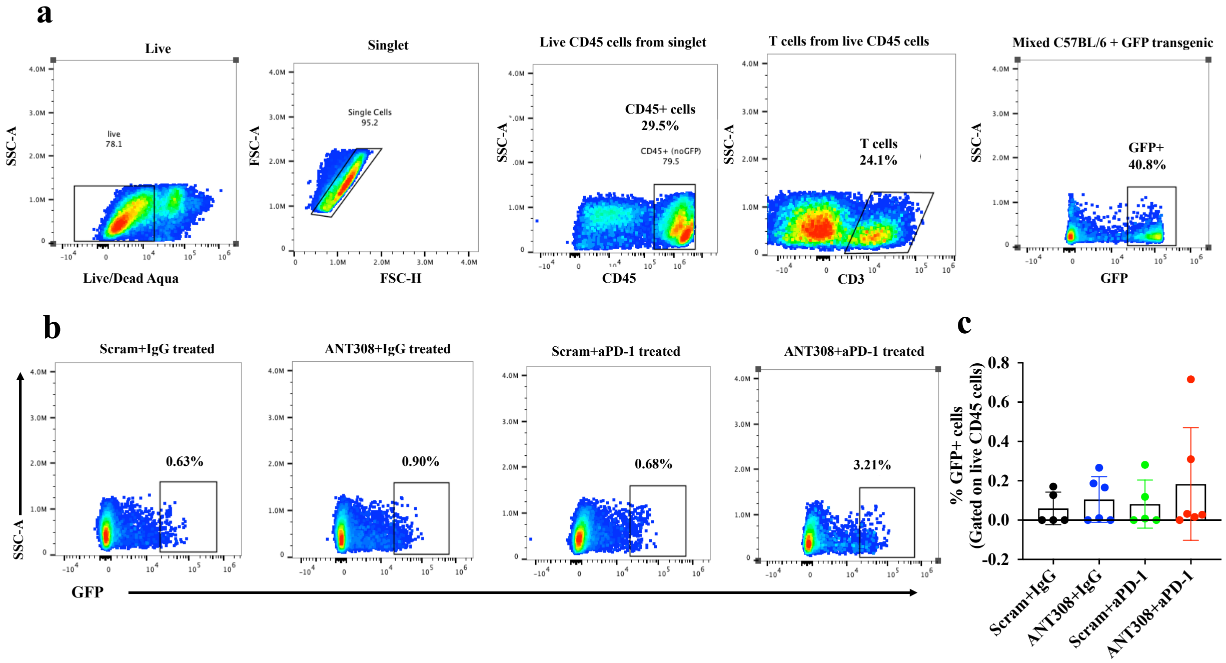

Supplementary Fig. 9. Increased frequency of GFP+ T cells in tumors of mice treated with the combination of VIP-R antagonist and anti-PD-1 as confirmed by flow cytometry. (a) Singlets from single-cell suspensions prepared from tumors of mice from Fig. 7a were gated by plotting forward scatter area (FSC-A) versus forward scatter height (FSC-H). Live CD45+ cells were gated by selecting CD45 positive, followed by gating for CD3 positive cells in CD3 versus SSC-A plots. GFP+ cells were then selected by gating GFP-positive cells based on mixed population of unstained splenocytes from naïve C57BL/6 mice and spleen samples from GFP transgenic mice. (b) Representative plot for CD3+GFP+ cells for four treatment groups (Scram+IgG, ANT308+IgG, Scram+anti-PD1, ANT308+ant-PD1) (c) Summary data from b showing percentage of GFP+ T cells over live CD45+ T cells (n=5 or 6 biologically independent samples). Percent GFP+ T cells were computed as the percentage of total CD3+GFP+ events divided by total live CD45+ events enumerated from FlowJo. Error bars show mean ± Standard deviation.

Supplementary Tables

Supplementary Table 1. Table depicting demographics of PDAC patients tested for serum VIP levels.

Patient #	Age	Gender	Race	Ethnicity
1	71	Female	Caucasian	Non-Hispanic
2	66	Male	Caucasian	Non-Hispanic
3	61	Female	African American	Non-Hispanic
4	75	Male	Caucasian	Hispanic
5	73	Male	Caucasian	Non-Hispanic
6	76	Male	Caucasian	Non-Hispanic
7	59	Female	Caucasian	Non-Hispanic
8	47	Female	African American	Non-Hispanic
9	69	Male	Caucasian	Non-Hispanic
10	69	Male	Caucasian	Non-Hispanic
11	64	Male	African American	Non-Hispanic
12	71	Female	Caucasian	Non-Hispanic
13	59	Female	African American	Non-Hispanic
14	69	Female	Caucasian	Non-Hispanic
15	71	Female	Caucasian	Non-Hispanic
16	67	Male	Caucasian	Non-Hispanic
17	69	Male	Caucasian	Non-Hispanic
18	52	Male	African American	Non-Hispanic
19	63	Male	Caucasian	Non-Hispanic

Supplementary Table 2. Details of fluorescent conjugated antibodies used in this study. (Abbreviations: Ag, antigen; CD, cluster of differentiation)

Target species	Target (Ag or CD#)	Fluorochrome	Clone	Vender	Catalog no.	Dilution
Human	CD3	PE-CF594	UCHT1	BD	562280	1:50
	CD4	APC-Cy7	RPA-T4	BD	557871	1:50
	CD8	Alexa Fluor 700	RPA-T8	BD	561453	1:50
	CD69	BV650	FN50	Biolegend	3190934	1:50
	CXCR4	PE-eFluor 610	12G5	eBioscience	610-9999-42	1:50
	PD-1	FITC	EH12.2H7	Biolegend	329904	1:50
	Tim-3	PE-Cy7	7D3	BD	345014	1:50
	Lag-3	APC	7H2C65	Biolegend	369212	1:50
	CD25	BV650	2A3	BD	562661	1:50
	FoxP3	PerCP-Cy5.5	236A/E7	BD	561493	1:30
Mouse	CD45	BV480	30-F11	BD	566095	1:50
	CD3	FITC	17A2	BD	561798	1:50
	CD4	Alexa Fluor 700	RM4-5	BD	557956	1:50
	CD8	PerCP-Cy5.5	53-6.7	BD	551162	1:50
	Ki67	PE-Cy7	16A8	Biolegend	652426	1:30
	CD25	APC-Cy7	3C7	Biolegend	101918	1:50
	FoxP3	PE	FJK16s	eBioscience	12-5773-82	1:30
	PD-1	BV785	29F,1A12	Biolegend	135225	1:50
	Tim-3	PE	B8.2C12	Biolegend	134004	1:50
	CXCR4	PE-CF594	2B11	BD	565019	1:50
	IFNg	FITC	XMG12	Biolegend	505806	1:30
	IL4	PE-Cy7	11B11	Biolegend	504118	1:30
	H-2Kb MuLV p15E	APC		MBL	TB-M507-2	1:10
In vivo MAb	PD-1		RMP1-14	BioXcell	BE0146	200µg (X4)
	Rat IgG2A Isotype control		2A3	BioXcell	BE0089	200µg (X4)
	CD4		GK1.5	BioXcell	BE0003-1	200µg (X5)
	CD8		Clone 2.43	BioXcell	BE0061	200µg (X5)
Other Antibodies	P-CREB (S133)	Alexa(R) 647	87G3	Cell Signaling	14001S	1:50
	VPAC1		SP234	Sigma	SAB5500193	1:500 (Western Blot)
	VPAC2		SP235	Sigma	SAB5500194	1:500 (Western Blot)

PD1	D4W2J	Cell Signaling	86163S	1:1000(Western Blot)
CTLA4	E1V6T	Cell Signaling	96399S	1:500 (Western Blot)
VIP	OTI5B5	Origene	CF806852	1:50 (Immunofluorescence)
Cytokeratin 19 (CK19)	EP1580Y	Abcam	ab52625	1:400 (Immunofluorescence)
CD4	EPR6855	Abcam	ab133616	mIHC
CD8	EP1150Y	Abcam	ab93278	mIHC
Ki67	SP6	Abcam	ab16667	mIHC

Supplementary Table 3. Statistical analysis showing synergistic effect between VIP-R antagonist and aPD-1 in orthotopic KPC-Luc model.

	Fractional total flux (FTF) relative to untreated controls[a]				
			Combination treatment		
a	VIP-R antagonist+IgG	Scrambled+aPD-1	Expected[c]	Observed	Ratio of expected FTF/observed[d] FTF
15	0.523	1.267	0.663	0.571	1.161
19	0.240	0.866	0.208	0.167	1.242
22	0.564	0.679	0.383	0.181	2.117

[a] FTF (mean tumor flux experimental)/(mean tumor flux scrambled+IgG)
[b] Day after tumor implantation
[c] (mean FTF of VIP-R antagonist+IgG) x (mean FTF of scrambled+aPD-1)
[d] Obtained by dividing the expected FTF by the observed FTF. A ratio of >1 indicates a synergistic effect, and a ratio <1 indicates a less than additive effect

Supplementary Methods

Sanger Sequencing

CRISPR-Cas9 VIPR2-KO panc02 cell pool was generated by Synthego (Redwood City, CA) with a confirmed efficiency of 98%. Guide sequence (UACCUCUCUGAUUCUCCGUU) was designed to target exon 2 of *VIPR2*. Single clones were selected with serial dilution in 96 well plates. After the colony was formed, DNA was extracted from single clone cell pellets using the QIAamp DNA Mini Kit from Qiagen (Cat# 51304). PCR was conducted using the following primers to amplify the region around gRNA: F: TTAGAAAGGTGAAGCGTTGGA (0.5 μM), R: TTTGCTGAAATCCCCACTGT (0.5 μM). The PCR reaction was performed with Promega GoTaq® Master Mixes (Cat# M7122) as follows: Hot start at 95 °C for 5min, denaturation at 95°C for 30s, annealing at 58°C for 45s, and extending at 72°C for 45s. The reaction was finished with an extra 5-min elongation at 72°C and a 20-min period at 4°C. The amplicon at 500bp was purified with a QIAquick Gel Extraction kit from Qiagen (Cat# 28704) and Sanger Sequencing was conducted at Genewiz/Azenta. Results were analyzed on. Clones with a model fit score of 1 and a knockout (KO) score of 100 were selected for further experiments.

RT-PCR

Total RNA was isolated with Trizol reagent (Thermo Fisher Scientific) and the first-strand cDNA was prepared using an AMV RNA PCR kit (TaKaRa) from 1 mg total RNA. PCR amplification for VPAC2 mRNA detection was carried out with an initial denaturing step at 95C for 5 min, then 35 cycles of PCR (95C for 30 s, 60C for 45 s, and 72C for 45 s) and a further extension at 72C for 10 min. The PCR products (Promega) and 50bp ladder (Thermo Fisher) were loaded on 4% of agarose gel and underwent electrophoresis in TBE buffer. UV system was applied for imaging.

Primers

GAPDH Forward AGGAGAGTGTTTCCTCGTCCC Reverse CAGATCCACGACGGACACAT

VPAC2 Forward ATGGACAGCAACTCGCCTCTCTTTAG Reverse GGAAGGAACCAACACATAACTCAAACAG

Supplementary References

1. Ward, J.M., M.J. Collins, Jr., and J.C. Parker, Naturally occurring mouse hepatitis virus infection in the nude mouse. *Lab. Anim. Sci.* **27**, 372-376 (1977).
2. Ward, J.M., et al., Chronic Active Hepatitis and Associated Liver-Tumors in Mice Caused by a Persistent Bacterial-Infection with a Novel Helicobacter Species. *Jnci-Journal of the National Cancer Institute* **86**,1222-1227 (1994).
