# Supplementary material for: Targeting vasoactive intestinal peptide-mediated signaling enhances response to immune checkpoint therapy in pancreatic ductal adenocarcinoma
Source: Nat Commun. 2022 Oct 27;13:6418. doi: 10.1038/s41467-022-34242-4 (PMC9613684; doi:10.1038/s41467-022-34242-4)
Supplement: Supplementary file 3 — Reporting Summary [file 41467_2022_34242_MOESM3_ESM.pdf]

nature portfolio

Corresponding author(s): Waller, Edmund K. & Ravindranathan, S.

Last updated by author(s): September 9th, 2022

Reporting Summary

Nature Portfolio wishes to improve the reproducibility of the work that we publish. This form provides structure for consistency and transparency in reporting. For further information on Nature Portfolio policies, see our Editorial Policies and the Editorial Policy Checklist.

Statistics

For all statistical analyses, confirm that the following items are present in the figure legend, table legend, main text, or Methods section.

n/a	Confirmed	
☐	☒	The exact sample size (*n*) for each experimental group/condition, given as a discrete number and unit of measurement
☐	☒	A statement on whether measurements were taken from distinct samples or whether the same sample was measured repeatedly
☐	☒	The statistical test(s) used AND whether they are one- or two-sided<br>*Only common tests should be described solely by name; describe more complex techniques in the Methods section.*
☐	☒	A description of all covariates tested
☐	☒	A description of any assumptions or corrections, such as tests of normality and adjustment for multiple comparisons
☐	☒	A full description of the statistical parameters including central tendency (e.g. means) or other basic estimates (e.g. regression coefficient) AND variation (e.g. standard deviation) or associated estimates of uncertainty (e.g. confidence intervals)
☐	☒	For null hypothesis testing, the test statistic (e.g. *F*, *t*, *r*) with confidence intervals, effect sizes, degrees of freedom and *P* value noted<br>*Give P values as exact values whenever suitable.*
☒	☐	For Bayesian analysis, information on the choice of priors and Markov chain Monte Carlo settings
☒	☐	For hierarchical and complex designs, identification of the appropriate level for tests and full reporting of outcomes
☒	☐	Estimates of effect sizes (e.g. Cohen's *d*, Pearson's *r*), indicating how they were calculated

Our web collection on statistics for biologists contains articles on many of the points above.

Software and code

Policy information about availability of computer code

Data collection	No software was used for data collection
Data analysis	FlowJo™ v10.8.1 was used to analyze fcs files for flow cytometry. TCR Sequencing data was analyzed using immunoSEQ® Analyzer. All statistical analysis was performed on GraphPad Prism software version 8.2.

For manuscripts utilizing custom algorithms or software that are central to the research but not yet described in published literature, software must be made available to editors and reviewers. We strongly encourage code deposition in a community repository (e.g. GitHub). See the Nature Portfolio guidelines for submitting code & software for further information.

Data

Policy information about availability of data

All manuscripts must include a data availability statement. This statement should provide the following information, where applicable:

- Accession codes, unique identifiers, or web links for publicly available datasets
- A description of any restrictions on data availability
- For clinical datasets or third party data, please ensure that the statement adheres to our policy

Nanostring data are available under GEO accession number (pendind confirmation receipt) and TCR sequencing data are available under GEO number (pending confirmation receipt) . VIP mRNA data are available on The Cancer Genome Atlas (TCGA). All data underlying individual figures within the manuscript are available under Source data file.

March 2021

Human research participants

Policy information about studies involving human research participants and Sex and Gender in Research.

Reporting on sex and gender	De-identified samples were used in the study. Both male and females were used for the study.
Population characteristics	Population characteristics for pancreatic cancer (PDAC) patients are provided in Supplementary Table 1. Ages range between 47 and 76 years with a mixed population of males and females. Plasma and histological samples of these patients were obtained from pancreatic cancer tissue repository. De-identified blood samples from healthy donors were obtained from sample acquisition bio-bank under an approved IRB protocol. Age for male or female healthy donor subjects range between 23 to 65 years. Apheresis products were purchased from Stem Cell Technologies.
Recruitment	Each healthy donor participants recruited under the IRB protocol were compensated with $30 amazon gift card for participation.
Ethics oversight	The Emory University Review Board approved the collection of blood samples from the participants.

Note that full information on the approval of the study protocol must also be provided in the manuscript.

Field-specific reporting

Please select the one below that is the best fit for your research. If you are not sure, read the appropriate sections before making your selection.

☒ Life sciences ☐ Behavioural & social sciences ☐ Ecological, evolutionary & environmental sciences

For a reference copy of the document with all sections, see nature.com/documents/nr-reporting-summary-flat.pdf

Life sciences study design

All studies must disclose on these points even when the disclosure is negative.

Sample size	The numbers of plasma samples tested were sufficient to detect a 3-fold difference in VIP levels between healthy individuals and PDAC patients with a standard deviation equal to twice the mean value of VIP in plasma from healthy individuals for a power of 0.8 and alpha of 0.05. T cells from 5 PDAC patients were expanded in vitro based upon the null hypothesis that experimental treatment would not significantly change the fraction of T cells expressing immune check-point molecules versus the alternative hypothesis that treatment would decrease the fraction of T cells expressing immune check-points by at least 50% with a power of 0.8, alpha of 0.05 using a paired t-test. Groups of.12 mice per treatment group were used for tumör experiments in which survival was an end-point based upon the null hypothesis that experimental treatment would not significantly change the friction of mice surviving beyond day 60 from 0% in the control group versus the alternative hypothesis that treatment would increase the faction of surviving mice to 40% with a power of 0.8, alpha of 0.05. Analyses of tumor samples and infiltrating immune cells in tumors were performed on a subset of mice used from survival experiments.
Data exclusions	No data was excluded.
Replication	Experiments were replicated at least two times with reproducible data for western blot. Results from representative experiments such as on micrographs were performed once as we had external validation with multiple mice or donors.
Randomization	Tumor-bearing mice were randomly allocated into treatment groups, with group composition adjusted (if needed) to ensure that mean tumor volumes at the time of treatment initiation were similar. For VIP plasma levels via enzyme immunoassay, patient samples and healthy donor samples were assigned randomly to the wells for the assay. For VIP immunofluorescence, cancer tissues and adjacent normal tissues were reviewed by a pathologist prior to staining for VIP. For T cell activation assays, healthy donors were chosen randomly. T cells isolated from individual healthy donors were activated with or without VIP-R antagonists or scrambled peptide. All samples were normalized to no peptide control for the individual donors.
Blinding	The investigator performing mouse experiments was not blinded as to treatment allocation as they were also responsible for preparing drugs to be injected. Blinding was not considered because survival end-point was defined by reaching maximum tumor volume volume of 500mm3 as approved by IACUC protocol. Pathologists scoring tissue sections for auto-immune pathology were blinded as to the treatment group from which the specimen was obtained.

Reporting for specific materials, systems and methods

We require information from authors about some types of materials, experimental systems and methods used in many studies. Here, indicate whether each material, system or method listed is relevant to your study. If you are not sure if a list item applies to your research, read the appropriate section before selecting a response.

Materials & experimental systems

n/a	Involved in the study
☐	☒ Antibodies
☐	☒ Eukaryotic cell lines
☒	☐ Palaeontology and archaeology
☐	☒ Animals and other organisms
☒	☐ Clinical data
☒	☐ Dual use research of concern

Methods

n/a	Involved in the study
☒	☐ ChIP-seq
☐	☒ Flow cytometry
☒	☐ MRI-based neuroimaging

Antibodies

Antibodies used	Catalog numbers with a list of antibodies used in the study are provided in Supplementary Table2.
Validation	Isotype controls or Fluorescent minus one (FMOs) were used as controls for flow cytometry analysis. Each lot of the antibody is quality control tested or validated by the manufacturers in multiple research applications.

Eukaryotic cell lines

Policy information about cell lines and Sex and Gender in Research

Cell line source(s)	MT5 and KPC-Luc cells were generous gifts from Dr. Tuveson (Cold Spring Harbor Laboratory, Cold Spring Harbor, NY) and Dr. Logsdon (MD Anderson Cancer Center, Houston, Texas), respectively and Panc02 cells were provided by Dr. Pilon-Thomas (H. Lee Moffitt Cancer Center, Tampa, FL). BXPC3, Panc1 and B16F10 were from ATCC (Manassass, VA) and SM1 were from Dr. Antoni Ribas (UCLA, Los Angeles, CA). Human pancreatic cancer associate stellate (PSC) cell line h-iPSC-PDAC-1 was generated and maintained as previously described in reference 78. The isolation of primary human fibroblasts from fresh samples of pancreatic cancer has been previously described in reference 79.
Authentication	Cell lines were purchased from ATCC with authentication as provided by ATCC or provided by our collaborators as described above. Cells were monitored for morphology as described by ATCC or by our collaborators. No other procedures were performed for authentication.
Mycoplasma contamination	All cell lines were tested negative for Mycoplasma contamination.
Commonly misidentified lines (See ICLAC register)	None

Animals and other research organisms

Policy information about studies involving animals; ARRIVE guidelines recommended for reporting animal research, and Sex and Gender in Research

Laboratory animals	Female or male C57BL/6, CD4KO (B6.129S2-Cd4tm1Mak/J) and CD8KO (B6.129S2-Cd8atm1Mak/J) were obtained from the Jackson Laboratory (Bar Harbor, ME) at 6-8 weeks of age. C57BL/6 background (strain designation: C57BL/6-Tg(Act-EGFP)C14-Y01-FM131 Osb) were a gift from Dr. Masaru Okabe (Osaka University, Osaka, Japan) and were bred and maintained at the Emory University Animal Care Facility (Atlanta, GA). A minimum of 5 mice or maximum of 20 mice per group was used.
Wild animals	No wild animals were used for the study
Reporting on sex	Male and female mice were used in equal proportion for the study. For example, each experimental group consists of 50% mice as males and 50% mice as females. We report no sex-based differences in our study. Data for this are presented in Supplementary Fig6d and e as well as are available under Source data file.
Field-collected samples	No field collected samples were used for the study
Ethics oversight	The study was approved under the PROTO201700866

Note that full information on the approval of the study protocol must also be provided in the manuscript.

Flow Cytometry

Plots

Confirm that:

☒ The axis labels state the marker and fluorochrome used (e.g. CD4-FITC).

☒ The axis scales are clearly visible. Include numbers along axes only for bottom left plot of group (a 'group' is an analysis of identical markers).

☒ All plots are contour plots with outliers or pseudocolor plots.

☒ A numerical value for number of cells or percentage (with statistics) is provided.

Methodology

Sample preparation	Harvested tumor tissues or tumor draining lymph nodes (TDLNs) from murine KPC-Luc or Panc02 bearing mice were cut into small pieces using a scalpel and treated triple enzyme digestion cocktail containing 10mg/ml collagenase, 1 mg/ml hyaluronidase and 200 mg/ml DNase in HBSS at 37°C for 20 minutes (TDLNs) or 1 hour (tumors) and vortexed every 15 minutes. The tissue pieces were then mechanically dissociated, washed, centrifuged, and passed through a 70µm nylon mesh filter to obtain a single cell suspension for staining and analysis via flow cytometry. For spleen samples, the single cell suspension obtained by mechanical dissociation, was passed through 70µm nylon mesh filter and depleted of red blood cells using ammonium chloride lysis buffer and washed twice. Blood samples were collected in tubes with 0.1ml diluted heparin (500 USP units/ml) followed by red blood cell depletion using ammonium chloride lysis buffer and washed twice. Approximately 1 million cells were collected and stained with Fixable Aqua live/dead stain from Thermo Fisher Scientific (Waltham, MA), followed by surface antibody staining for thirty minutes on ice. To identify tumor specific T cells, cells were stained with APC conjugated MHC Tetramer H-2kb MuLV p15E for fourty-five minutes at room temperature (MBL International Corporation) prior to surface staining. For intracellular cytokine expression staining, cells were incubated with leukocyte activation cocktail (BD) for 5 hours, then stained with antibodies listed in Supplementary Table 1.
Instrument	FACS files were acquired with a FACS Aria cytometer (Beckon Dickinson, San Jose, CA) or an Aurora cytometer (Cytek Biosciences, Inc, Fremont, CA)
Software	FlowJo™ v10.8.1
Cell population abundance	Single cell suspensions of tumor were subjected to magnetic T cell isolation using EasySepTM Mouse CD90.2 Positive selection kit II from Stem Cell Technologies. The purity of T cell following isolation was 85%.
Gating strategy	Low side scatter was used to gate for T cell population, followed by singlet gating using FSC-A vs FSC-H. Live cells were gated using negative population for live-dead Aqua stain. Positive gates were determined using Isotype controls or fluorescent minus one (FMO) control for each antibody for every single runs. Schematic Gating Strategies are provided in figure4c supplementary figure3-5 and supplementary figure 9.

☒ Tick this box to confirm that a figure exemplifying the gating strategy is provided in the Supplementary Information.
